# Some aspects of formation and tribological properties of silver nanodumbbells

**DOI:** 10.1186/1556-276X-9-186

**Published:** 2014-04-21

**Authors:** Boris Polyakov, Sergei Vlassov, Leonid M Dorogin, Natalia Novoselska, Jelena Butikova, Mikk Antsov, Sven Oras, Runno Lohmus, Ilmar Kink

**Affiliations:** 1 Institute of Solid State Physics, University of Latvia, Kengaraga str. 8, Riga LV-1063, Latvia; 2 Institute of Physics, University of Tartu, Riia str. 142, Tartu 51014, Estonia; 3 Estonian Nanotechnology Competence Center, Riia str. 142, Tartu 51014, Estonia; 4 I.I.Mechnikov Odessa National University, Dvoryanskaya str. 2, Odessa 65082, Ukraine

**Keywords:** Silver nanowires, Nanomanipulation, Tribology

## Abstract

In this paper, metal nanodumbbells (NDs) formed by laser-induced melting of Ag nanowires (NWs) on an oxidized silicon substrate and their tribological properties are investigated. The mechanism of ND formation is proposed and illustrated with finite element method simulations. Tribological measurements consist in controllable real-time manipulation of NDs inside a scanning electron microscope (SEM) with simultaneous force registration. The geometry of NDs enables to distinguish between different types of motion, i.e. rolling, sliding and rotation. Real contact areas are calculated from the traces left after the displacement of NDs and compared to the contact areas predicted by the contact mechanics and frozen droplet models.

**PACS:**

81.07.-b; 62.25.-g; 62.23.Hj

## Background

Metal nanoparticles (NPs) are well-known objects for tribological studies and nanomanipulation experiments [1]. The majority of studies had been performed on NPs assumed to be spherically shaped, while significantly less number of works was dedicated to nonspherical NPs [2-5]. Taking into account the fact that the friction force at the nanoscale is proportional to the contact area [6], it is important to know the exact geometry of NPs for correct calculation of their contact area. However, in the case of spherical NPs, it is difficult to distinguish between sliding, rolling and rotating motions. Therefore, an elongated object (e.g. nanowire or nanorod) could be more suitable for revealing different regimes of motion in tribological tests. However, due to increased contact area (and static friction), the manipulation of elongated structures can be problematic. For example, the displacement of CuO nanowires (NWs) on a smooth silicon substrate is almost impossible without damaging and breaking of NWs [7].

Metal NWs (especially Ag NWs) are a perspective class of materials for transparent conductive electrodes, intensively investigated during the last few years [8,9]. Optical welding of NW percolating networks is a fast and cost-effective method of improving the conductivity of an electrode by improving wire-to-wire contact resistance [10]. NW-to-substrate adhesion after optical or laser processing is a key parameter of NW-based electrode operation.

Laser-induced melting of metal nanostructures is an intriguing phenomenon studied by several research groups. Habenicht et al. described laser-induced melting, dewetting and ejection (‘jumping’) of Au nanoparticles formed from triangular nanostructures on HOPG substrate [11]. The driving mechanism of NP ejection was minimization of surface energy of the liquid droplet, and the NP ejection velocity was proportional to the energy of laser pulse. In spite of the small time span of melting, ejection and solidification processes (ns), some NPs were frozen in different stages of dewetting and ejection. This phenomenon was analysed and numerically simulated by Afkhami and Kondic [12]. Laser-induced melting of Ag NWs was recently investigated by Liu et al. [13]. They analysed the distribution of electric field and melting patterns along the length of a NW. Maximal field is concentrated on the ends of a NW, promoting melting of the ends of the NW. At relatively small laser pulse energy, spheroid-like structures are formed on the ends of NWs. The resulting nanostructure resembles a ‘dumbbell’ that hereafter will be referred as a nanodumbbell (ND). At higher pulse energy, spherical particles can detach from the NW, or even the whole NW can be melted into the separated spherical NPs due to Rayleigh-Plateau instability [14].

A ND can be roughly considered as two spheroidal NPs connected by a NW. A ND is a novel and attractive object for nanotribological studies. If the distance between the rounded ends of a NW is short enough, the dumbbell might rest on the rounded ends mainly. Thus, the end bulbs of a ND ensure a relatively small contact area, reduced adhesion and static friction compared to those of intact NWs. Therefore, NDs can be easily manipulated, and different types of motion can be distinguished (sliding, rolling, rotation). However, subsequent analysis and interpretation of experimental data can be complicated. In particular, correct determination of the contact area of NDs is a nontrivial problem. Conventional contact mechanics models developed for solid spherical particles cannot be applied for calculation of the ND contact area. This is due to the physics of ND formation that involves melting and solidifying of NPs on their ends, and this is needed to be taken into account.

In this work, we studied formation and tribological properties of Ag NDs produced by laser processing of corresponding metal NWs on an oxidized silicon surface. Detachment of the ND central part was discussed and analysed using finite element method simulations. Contact areas and static friction of end bulbs of NDs were investigated experimentally and analysed theoretically. NDs were manipulated on oxidized silicon wafers inside a scanning electron microscope (SEM) with simultaneous force recording. Different motion types of NDs were observed during the experiment. To the best of our knowledge, metal NDs were used for nanomanipulations for the first time.

## Methods

Ag NWs of 120 nm in diameter were purchased from Blue Nano (Charlotte, NC, USA). The nanowires were deposited on an oxidized silicon wafer substrate (cut from a 3-in. wafer, 10^-3^ Ω cm, 50 nm thermal SiO_2_, Semiconductor Wafer, Inc*.*, Hsinchu, Taiwan) from solution. For laser treatment of the samples, the second harmonic (532 nm) of Nd:YAG laser (Ekspla NL-200, Vilnius, Lithuania) with a pulse duration of 9 ns and a repetition rate of 500 Hz was used. The beam diameter was 0.6 mm, and the laser pulse energy was approximately 0.9 mJ. After laser treatment, Au and Ag NDs were examined in a transmission electron microscope (Tecnai GF20, FEI, Hillsboro, OR, USA).

The experimental set-up comprised of a 3D nanopositioner (SLC-1720-S, SmarAct, Oldenburg, Germany) equipped with a self-made force sensor installed inside a SEM (Vega-II SBU, TESCAN, Brno, Czech Republic; typical chamber vacuum 3 × 10^-4^ mbar). High-resolution images of NDs and traces left after displacement of NDs were taken inside FEI Helios Nanolab SEM. The force sensor was made by gluing a commercial atomic force microscope (AFM) cantilever with a sharp tip (Nanosensor ATEC-CONT cantilevers, Neuchatel, Switzerland, *C* = 0.2 N/m) to one of the prongs of a commercially available quartz tuning fork (QTF). The signal from the QTF was amplified by a lock-in amplifier (SR830, Stanford Research Systems, Sunnyvale, CA, USA) and recorded through the ADC-DAC card (NI PCI-6036E, National Instruments, Austin, TX, USA). The typical values of the driving voltage were 20 to 50 mV, and the corresponding tip oscillation amplitude was in the order of 100 nm. The tip oscillated parallel to the sample surface, i.e. in the shear mode.

During the experiments, the tip was positioned at about the half height of a ND above the substrate surface. Each manipulation experiment started with a displacement of the ND from its initial position by an abrupt tip motion to reduce the initial adhesion. Initial displacement was followed by controlled manipulation of the ND by pushing it with the AFM tip with simultaneous force recording. During the manipulation, the tip moved parallel to the surface along a straight line without feedback loop. The point of the tip contact with ND was varied to investigate different scenarios of ND behaviour. More details about the nanomanipulation technique can be found in [15].

The Solid Mechanics module in COMSOL Multiphysics (version 4.3b) was used to build a stationary physics model of a deflected dumbbell resting on a flat substrate. The material properties of Ag were taken from the COMSOL material library; only Young's modulus was added manually, with the value 83 GPa.

## Results and discussion

### ND formation process

**Figure 1:**
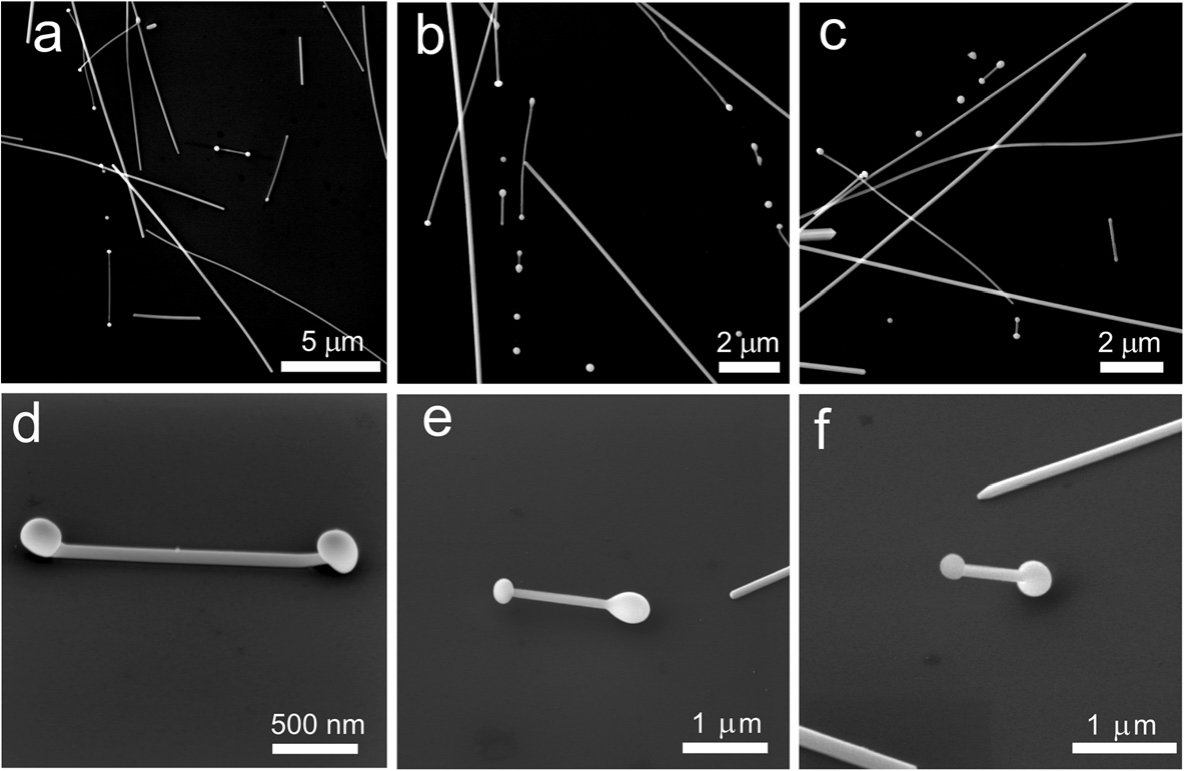
**Nanostructures produced by laser processing of Ag NWs.** NWs with end bulb, NDs of different length and spherical particles are typically produced **(a-c)**. Partial rising of NDs from the substrate, imaged at 52° SEM stage tilt **(d)**. Central part of Ag NDs is completely suspended, imaged at 45° **(e)**. Ag ND rests on one bulb only, imaged at 45° **(f)**.

SEM investigation revealed that after laser processing, most of the Ag NWs have rounded ends (end bulbs), and a large number of spherical NPs and some NDs were produced (Figure 1). Similar nanostructures can be produced by laser processing of Au NWs (Additional file 1: Figure S1). ND formation is a complicated dynamic process, which involves extreme temperature gradients, and includes rapid heating and melting of the ends of NWs, contraction of liquid droplets into spheroidal bulbs and followed by rapid solidification.

**Figure 2:**
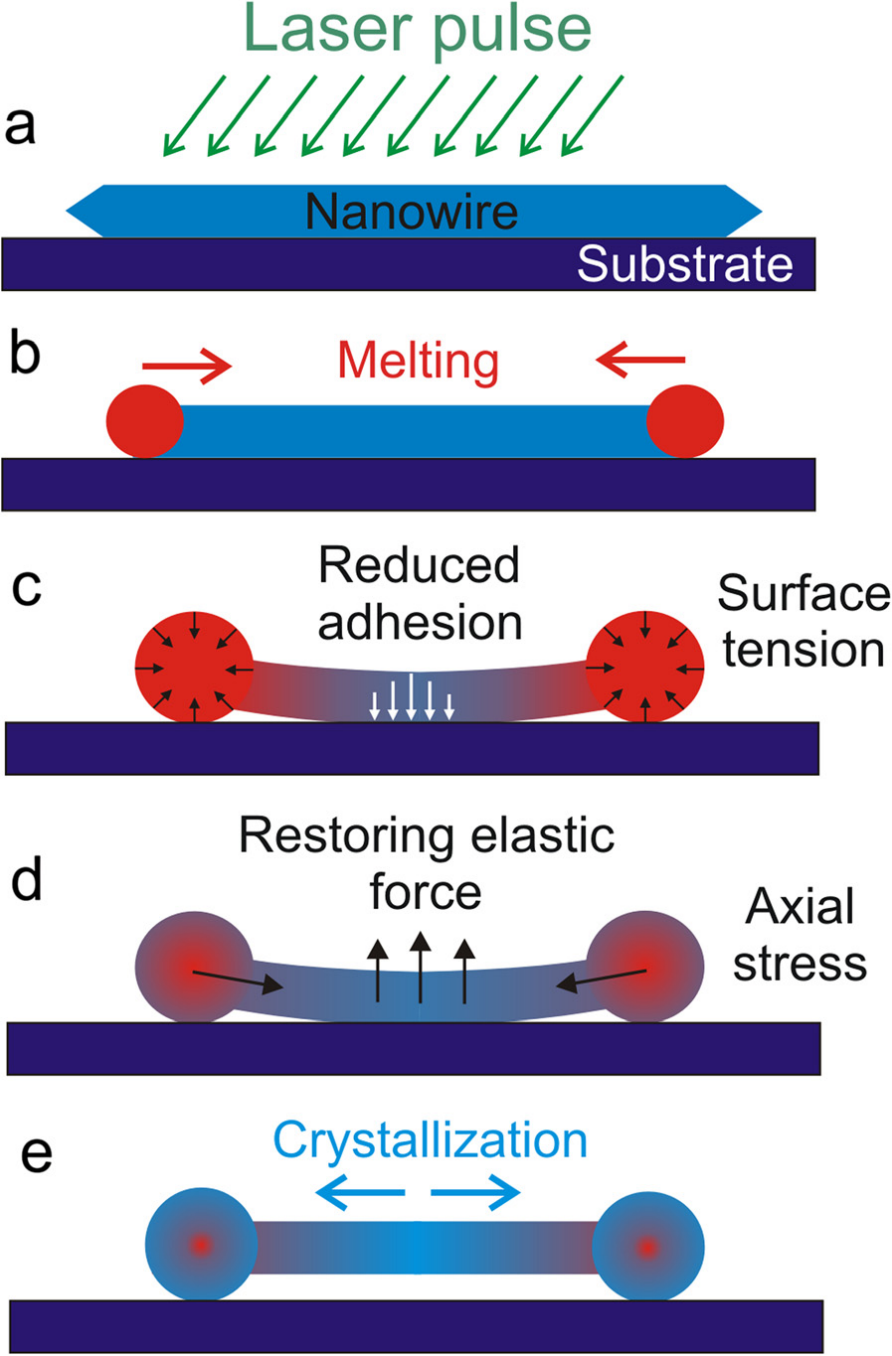
**Schematics of ND formation.** Laser treatment **(a)**. NW ends are melting, and the NW length decreases **(b)**. Surface tension detaches a part of NW near the end bulbs from the substrate **(c)**. Crystallization and elastic straightening of NW connecting two end bulbs of ND **(d)**. Complete solidification of ND **(e)**.

Let us propose a mechanism of ND formation using SEM images of NDs frozen at different stages of formation. After absorption of laser pulse energy, a NW starts to melt; liquid droplets grow in volume and move towards the centre of a NW (Figure 2a,b). Surface tension tends to minimize the surface area of a droplet and makes it spherical. The temperature of the parts of a NW close to the liquid bulbs approaches the melting point, causing a local decrease of Young's modulus and resulting in the detachment of the parts from the substrate pulled by the growing droplet (Figure 2c). Adhesion of the central part of a NW resting on the substrate is significantly reduced due to inverse dependence of surface free energy on temperature [16]. However, the temperature in the central part of a NW is below the melting point, since the NW preserves its original crystalline structure (Additional file 1: Figure S2). When the ND is cooled down, the middle part becomes a crystallization nucleus and defines the epitaxial crystallization of the melted part of the wire towards the end bulbs. After solidification, there is an elastic stress tending to restore the straight profile of the bent part connecting two bulbs. Restoring force is also enhanced by the axial stress that originated from the thermal contraction of cooling wire (Figure 2d). If the part of the NW adhered to the substrate is short enough, and adhesion force is less than restoring elastic forces, the middle part of the NW can get detached from the substrate, and the ND will rest on the end bulbs only (Figure 2e). It is worth to note that in spite of rapid cooling, the end bulbs are crystalline as it was demonstrated by Liu et al. [13].

SEM observations show that some NWs were completely removed from the substrate by laser processing, where former positions of NWs can be identified as dark ‘shadows’ on the surface of the substrate (Additional file 1: Figure S3). Examination at 45° sample tilt reveals that a number of NDs contact the substrate by one end only (Figure 1f). Complete detachment is likely connected to the ejection of the liquid droplets described by Habenicht et al. [11]. The exact mechanism of melting and complete detachment of NWs is rather complex and requires advanced computer simulations [17,18].

In order to support the proposed mechanism of ND formation, let us consider a rough estimation of the balance of forces involved on the stages of separation of ND from the substrate: adhesion of the NW, elastic force of the bent NW pulled by the bulbs and thermally induced stress in the NW.

Contact pressure caused by adhesion between the facet of the NW and the underlying substrate can be estimated as [19]

(1) $$P=\frac{A}{6\pi D^{3}},$$

where *A* is the Hamaker constant for the Ag/SiO_2_ system and *D* is the cutoff distance [19]. The Hamaker constant for the system can be approximated as $A=\sqrt{A_{\mathrm{Ag}}A_{\mathrm{SiO}2}}$, where *A*_Ag_ is the Hamaker constant of silver and *A*_SiO2_ is the same for SiO_2_, with values 3.72 × 10^-19^ and 0.62 × 10^-19^ J, respectively, and the cutoff distance is approximately *D* ≈ 0.2 nm [19]. Using Equation 1, the calculated contact pressure for the system is approximately 1 GPa, which is the minimal pressure necessary to separate the contacting bodies.

**Figure 3:**
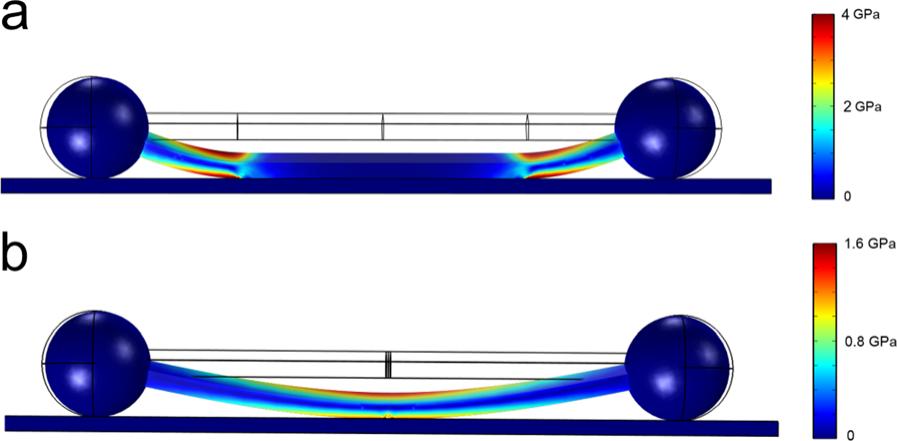
**FEM simulations of elastic behavior of a ND adhered to a substrate.** The bulb radius is 175 nm, total wire length 2 μm, and the wire cross section is pentagonal of 100 nm in diameter. **(a)** First case - adhered part length 1 μm. **(b)** Second case - adhered part length 10 nm.

A finite element method (FEM) simulation was used to study the elastic behaviour of an Ag dumbbell structure interacting with a flat substrate (more details in Additional file 1: Figure S4). The model consisted of a dumbbell-like geometry resting on a flat rectangular block. The first case (Figure 3a) describes the earlier stage of dumbbell formation; the length of the adhered part was chosen to be 1 μm long. The second case (Figure 3b) depicts a later stage of dumbbell formation, where most of the wire between the balls is detached (the length of the adhered part is 10 nm). In the vicinity of the interface separation edge, the elastic stresses are concentrated and may reach 0.5 to 4 GPa, which can be sufficient to induce interface separation. Note that the stress decreases with the decrease of the length of the adhered part; thus, only relatively short NDs are able to detach from the substrate completely.

The thermal stresses induced by contraction of the NW due to cooling may play a significant role in the interface separation as well. The thermal strain _th_ can be estimated from the following equation:

(2) $$\epsilon_{\mathrm{th}}=\alpha_{\mathrm{Ag}}\Delta T,$$

where *α*_Ag_ is the thermal expansion coefficient of silver and Δ*T* is the difference of the initial and final temperatures. The thermal expansion coefficient of bulk silver is 19.7 × 10^-6^/K [20], and considering the temperature difference of 680 K, the strain for such a process is approximately 1.34%. Calculating the thermal stress by *σ*_th_ = *E*_Ag__th_, where *E* is Young's modulus for silver (*E*_Ag_ ≈ 83 GPa), one yields *σ*_th_ ≈ 1.1 GPa. As the result of superposition of the elastic stress of bent NW and thermal stress, interface separation takes place similarly to crack propagation.

### Contact area and static friction

The contact area, as well as friction between the end bulbs and the substrate, will strongly depend on the shape of the bulbs. According to the experimental observations, the end bulbs of the NDs have an ellipsoidal shape that is close to prolate spheroid with the semi-axes *R*_1_ and *R*_2_. For purposes of simplicity, we will use spherical ball approximation, justified by the ratio *R*_1_/*R*_2_ ~ 1. Thus, the effective radius $R_{e}=\sqrt{R_{1}\cdot R_{2}}$ will only be used.

The real shape of the bulb is a result of the dynamic interplay of surface tension and adhesion forces in a liquid droplet followed by solidification. In this regard, two boundary cases can be considered. In the first case, named frozen droplet model (FDM), a molten bulb solidifies in contact with the substrate and takes the shape of truncated ellipsoid defined by the contact angle of a liquid droplet with the solid substrate. The estimation of the contact area *A* is obtained from geometrical consideration for a spheroid of radius *R*_e_ and a cutting plane of the contact:

(3) $$A=\pi \cdot R_{e}^{2}\cdot \sin^{2}\Theta ,$$

where *Θ* is the contact angle for the Ag/SiO_2_ interface.

In another scenario, the molten structure detaches from the substrate, as was shown in several works [11,17], and solidifies before contacting the substrate again (Figure 1f). The bulb shape will be close to the sphere or ellipsoid, and the contact will be governed by adhesion and elastic forces. Such situation can also occur when ND with frozen droplet-shaped bulbs is displaced from its initial position and rolled to the ‘rounded’ side of the bulbs.

The contact area of the sphere-on-plane can be calculated on the basis of continuum elasticity models for deformable spheres such as JKR [21] or DMT-M model [22], which also gives a good approximation for ellipsoids providing *R*_1_/*R*_2_ ~ 1 [19]. According to Tabor [23], the choice of the most suitable model is determined by the parameter

(4) $$\eta ={\left(\frac{16R_{e}\gamma^{2}}{9K^{2}z_{0}^{3}}\right)}^{1/3},$$

where *γ* is the work of adhesion and *z*_0_ is the equilibrium spacing for the Lennard-Jones potential of the surfaces. *K* is the combined elastic modulus of the sphere and substrate, defined as

(5) $$K=\frac{4}{3}\left(\frac{1-{\nu_{1}}^{2}}{E_{1}}+\frac{1-{\nu_{2}}^{2}}{E_{2}}\right),$$

in which *ν*_1,2_ and *E*_1,2_ are the Poisson ratios and Young moduli of the substrate and sphere, respectively. For small *η*, the DMT-M theory is more appropriate [24] and will be used below. According to the DMT-M model, the contact area *A*_DMT_ of the sphere on a flat surface is

(6) $$A_{\mathrm{DMT}}=\pi {\left(\frac{2\pi \gamma }{K}\right)}^{2/3}R_{e}^{4/3},$$

Friction force can be expressed as the following simple form:

(7) $$F_{\mathrm{friction}}=\tau \cdot A,$$

where *τ* is the interfacial shear stress/strength and *A* is the contact area [25]. The shear strength is defined as an ultimate shear stress *τ* before the object is displaced and can be estimated using the relation *τ*_theo_ = G* / *Z*, where *ν* is Poisson's ratio and G* = [(2 - *ν*_1_) / *G*_1_ + (2 - *ν*_2_) / *G*_2_]^-1^[25,26]. *Z* is an empirical material-dependent coefficient ranging from 5 to 30 [27]. Taking *Z* = 15 as the typical value for most metals [27], theoretical shear strength for Ag equals *τ* ≈ 0.59 GPa.

### Real-time manipulations

Nanomanipulation technique inside SEM with simultaneous force registration was used to control the applicability of FDM and DMT-M models for description of ND contact with the substrate surface experimentally. The experiment has shown that in most cases, the end bulbs of NDs ensure a relatively small contact area and therefore reduced adhesion and friction force. For comparison, displacement of untreated uniform Ag NWs on a flat silicon substrate was almost impossible without severe damage and plastic deformation of NW (Additional file 1: Figure S5).

NDs exhibited several regimes of motion in manipulation experiments. The most common scenario was rotation of the ND around one of its ends. Long-range rolling of Ag NDs was rarely observed, while rolling up to approximately 90° was registered frequently. In some cases, one end of ND was losing contact with the substrate surface, and ND rotated around the adhered end out of the substrate plane. In a few cases, static friction was high enough to keep one of the ends fixed, which led to plastic deformation of the ND during manipulation (Additional file 1: Figure S6).

**Figure 4:**
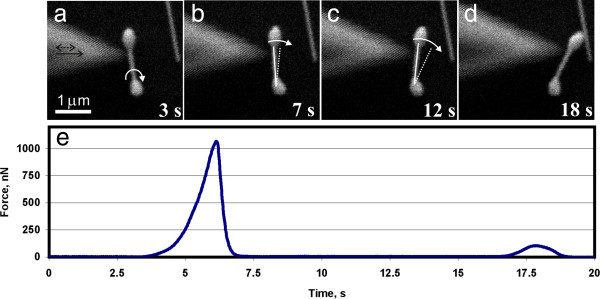
**Manipulation of an Ag ND.** The solid black arrow indicates the direction of the tip movement, and the dashed black arrow shows the direction of oscillation of the tip **(a)**. The ND rolls over approximately 90° **(a, b)**, then rotates around one of its bulbs **(b-d)** and finally runs into a NW **(d)**. White arrows indicate the type of motion. Corresponding tip-dumbbell interaction force in time was recorded by a QTF sensor **(e)**.

Typical experiment of ND manipulation is shown in Figure 4. After overcoming the static friction force *F*_st_ ≈ 1 μN, ND first rolled over (Figure 4a,b) and then rotated around one of the ends at almost zero force until it ran into neighbouring NWs (Figure 4c,d). Kinetic friction during ND rotation was below the detection limit. The huge difference between the static and kinetic friction agrees with our previous work performed on Au NPs [15].

**Figure 5:**
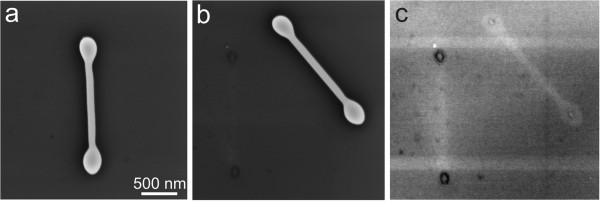
**Traces after ND displacement indicating the contact area.** Intact ND **(a)**. First displacement (without rolling) of the ND **(b)**. Second displacement of the ND, contrast-enhanced to reveal ‘traces’ (black elliptical regions correspond to the former position of ND bulbs) **(c)**.

**Figure 6:**
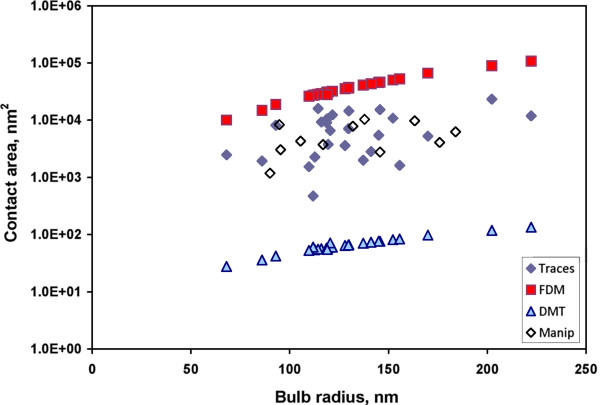
**Comparison of contact areas calculated from experimentally measured friction force and trace areas.** Areas of experimentally observed ND traces (Traces), calculated area from friction measurements (Manip), and contact areas calculated by frozen droplet (FDM) and DMT-M (DMT) models. The used parameters are as follows: Θ = 123.8° (contact angle of Ag/SiO_2_) [27], *ν*_1_ = 0.17 (Poisson’s ratio of SiO_2_) [28], *ν*_2_ = 0.36 (Poisson’s ratio of Ag) [28], *E*_1_ = 71.7 (Young’s modulus of SiO_2_, GPa) [28], *E*_1_ = *E*_Ag_ = 82.5 (Young’s modulus of Ag, GPa) [28], *γ* = 50 × 10^-3^ (the work of adhesion, J/m^2^) [21], *z*_0_ = 0.3 (equilibrium spacing for the Lennard-Jones potential of the surfaces, nm) [29], *K* = 55.4 (combined elastic modulus, GPa), *η* = 0.2 (Tabor’s coefficient).

In general, static friction forces measured for ten NDs were scattered from 200 to 1,750 nN. To find the reason for such large variation of static friction force values of manipulated NDs, we studied contact areas of 24 NDs after displacement using residual traces inside a high-resolution SEM, (Figure 5) and compared these experimental values with calculated ones. Here we need to mention that physical reasons behind the residual traces are not yet clear; however, the visible trace area can be considered proportional to the real contact area. To prove this assumption, we manipulated untreated Ag NWs, which have a well-defined pentagonal cross section [28]. The width of the traces left after displacement corresponded to the width of one pentagon facet (Additional file 1: Figure S4). In the next step, we compared contact areas calculated from experimentally measured friction force for one set of NDs using Equation 7 (Figure 6, *Manip*) and trace areas for another set of NDs (Figure 6, *Traces*). As it can be observed from Figure 6, there is good agreement between both contact areas.

Experimentally observed trace areas remained after ND displacement; contact areas calculated for the same NDs according to the FDM (Equation 3) and DMT (Equation 6) approaches using radii of ND end bulbs, measured in SEM, are shown in Figure 6. It is evident that experimental results obtained by trace observations are closer to values of contact area calculated by FDM than to those by the DMT-M model (Figure 6). It means that the end bulbs of these NDs are not perfect spheroids, but truncated ones solidified in the contact with the substrate. However, the obtained experimental values are still lower than FDM predicts. The possible reasons for FDM to overestimate the contact area are as follows: (1) the equilibrium shape of the droplet may differ significantly from the truncated spheroid, (2) the droplet solidifies before reaching the equilibrium shape, (3) it is possible that the contact angle of the substrate surface with liquid metal nanodroplets is larger than the contact angle of that with macroscopic droplets (135° to 150° instead of 123.8°).

A phenomenon directly related to variations in friction force and contact area is a temporal dependence of contact area or aging [15,30]. The force required to displace NDs was inversely proportional to the time intervals between the manipulation events. Figure 5c demonstrates the traces left after the first and the second displacement of the same ND (time interval of a few minutes). The area of the first pair of traces is approximately 9.03 × 10^3^ and 10.82 × 10^3^ nm^2^ and only approximately 2.63 × 10^3^ and 2.62 × 10^3^ nm^2^ for the second pair of traces. Analysis of the shape of this ND before and after displacement provides evidence that ND was displaced by sliding and rotation only. Therefore, the decrease of the contact area in this case cannot be explained by rolling of the ND onto the more spherical side of the end bulbs. Possible explanation of contact aging is diffusion of metal atoms, which can be accelerated by local heating or migration of electrons caused by the electron beam of SEM. However, detailed analysis of the contact aging phenomenon is out of the scope of this article.

## Conclusions

It was demonstrated that metal NDs are attractive objects for nanomanipulation and nanotribology. Formation of metal ND on the substrate from a NW under laser beam radiation is a complex process. The final configuration of a ND is a result of the interplay between the intrinsic effects (i.e. melting, crystallization, effect of thermal stress, elastic forces) and adhesion during the separation of the NW from the substrate. The experimental study showed reduced contact area and adhesion of NDs in comparison to intact NWs. The geometry of NDs enabled to study different regimes of motions in manipulation experiments, i.e. sliding, rolling and rotation. Contact areas and static friction forces of NDs were measured and compared to the DMT-M and FDM contact models.

## Abbreviations

FDM: frozen droplet model; FEM: finite element method; ND: nanodumbbell; NP: nanoparticle; NW: nanowire; SEM: scanning electron microscope.

## Competing interests

The authors declare that they have no competing interests.

## Authors’ contributions

BP, SV and LD planned the experiment and drafted and revised the manuscript. BP, SV and SO carried out all experiments. LD, NN and SO analysed the results and processed the data. JB performed the laser treatment of the samples and revised the manuscript. MA carried out the Comsol simulations. IK and RL supervised the research, coordinated the study and revised the manuscript. All authors have read and approved the final manuscript.

## Supplementary Material

Additional file 1 **Supplementary materials.** The file contains Figures S1 to S6 and discussion on COMSOL simulations. Click here for file
