# Supplementary material for: Some aspects of formation and tribological properties of silver nanodumbbells
Source: Nanoscale Res Lett. 2014 Apr 21;9(1):186. doi: 10.1186/1556-276X-9-186 (PMC4022052; doi:10.1186/1556-276X-9-186)
Supplement: Additional file 1 — Supplementary materials. The file contains Figures S1 to S6 and discussion on COMSOL simulations. [file 1556-276X-9-186-S1.pdf]

Some aspects of formation and tribological properties of silver nanodumbbells

B. Polyakov[a*], *S. Vlassov*[a], *L. Dorogin*[b,c], *N. Novoselska*[d*], *J. Butikova*[a], *M. Antsov*[b,c], *S. Oras*[b,c]
I. Kink[b,c], *R. Lohmus*[b,c]

*Corresponding Authors
boris.polyakov@cfi.lu.lv
natalia.novoselska@gmail.com

a Institute of Solid State Physics, University of Latvia, 8 Kengaraga Street, Riga, LV-1063, Latvia
b Institute of Physics, University of Tartu, Riia 142, 51014, Tartu, Estonia
c Estonian Nanotechnology Competence Center, Riia 142, 51014, Tartu, Estonia
d I.I.Mechnikov Odessa National University, Dvoryanskaya str., 2,Odessa, 65082, Ukraine

Supplementary materials

Figure S1.

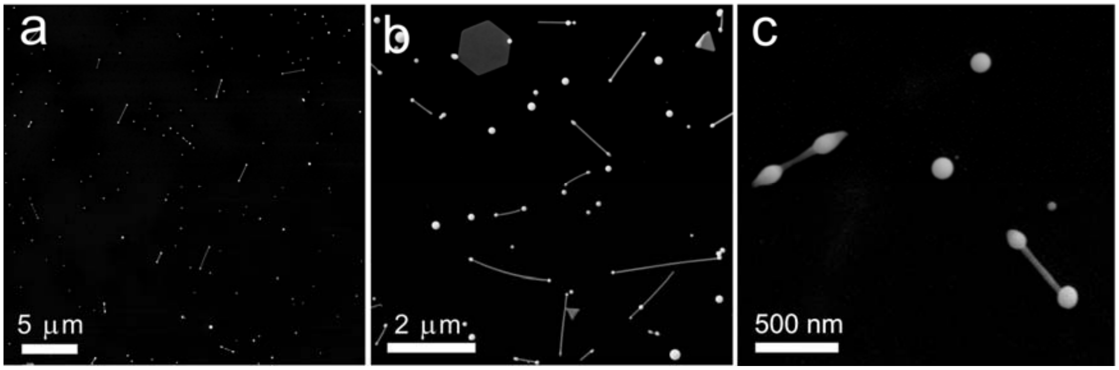

Au NDs produced from Au NWs by pulsed laser treatment.

Figure S2.

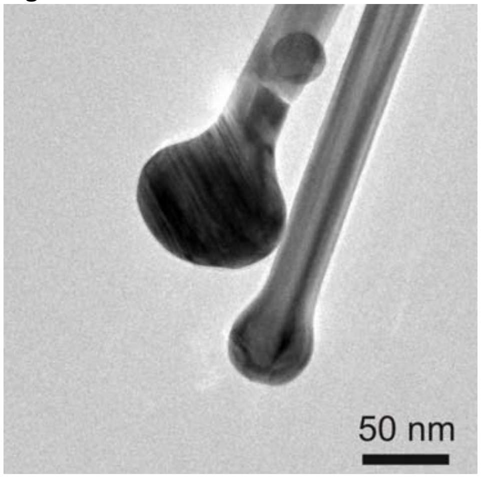

TEM image of Ag NDs.

Figure S3.

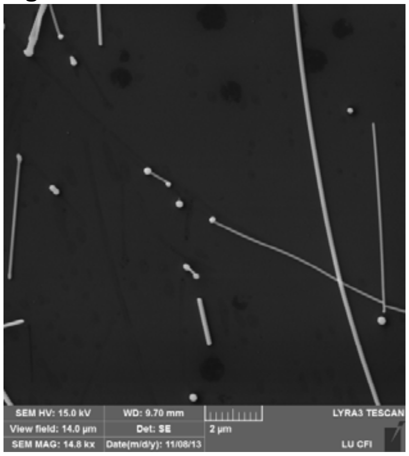

Some Ag NWs were completely removed from the substrate by laser processing, where the former positions of NWs can be identified as dark “shadows” on the surface of the substrate.

COMSOL simulations

A finite element method (FEM) simulation is used to study the elastic behaviour of an Ag dumbbell structure interacting with a flat substrate. The model consisted of a dumbbell-like geometry resting on a flat rectangular block. The distance between the two balls forming the dumbbell was kept constant at 2 microns. For determining the elastic stress distribution in a dumbbell during its formation, two different systems were simulated: 1) elastic stress distribution in ND having end bulb radius 175 nm and adhered part 1000 and 10 nm (fig.3), and 2) elastic force dependence on bulb radius in range 55-275 nm (fig. S3). The material properties for Ag were taken from the Comsol material library, only the Young’s modulus was added manually, with the value 83 GPa. The mesh for the geometry/simulation was created with two different distributions. In the vicinity of the deflected boundary edges, the mesh was made denser, consisting of maximum and minimum elements with values 1e-8 m and 5.2e-10 m, respectively. The mesh for the rest of the geometry consisted of elements, width maximum and minimum element sizes being 2.6e^-7 m and 2.68e^-8 m, respectively. For both cases contact boundary was set between the block and the balls with zero friction. The displacement freedom of the system was fixed in certain directions: the whole system was fixed in the x direction (perpendicular to the displacement of the pentagonal bridge and perpendicular to the parallel axis (z) of the bridge), the displaced boundary was fixed in the x and z direction (z is parallel to the pentagonal bridge). The whole system was free in the y direction, which is the normal of the surface.
For determining the elastic forces, the above described system was simulated over the radius of the two balls ranging from 55-275 nm. A 10 nm wide area in the centre of a side of the pentagonal beam facing the underlying block was held against the surface during each simulation step by a boundary condition, which resulted in a certain elastic force for each deformed (deflected) shape of the beam. The result can be seen in figure S3, where the force vs. radius is depicted.

Figure S4.

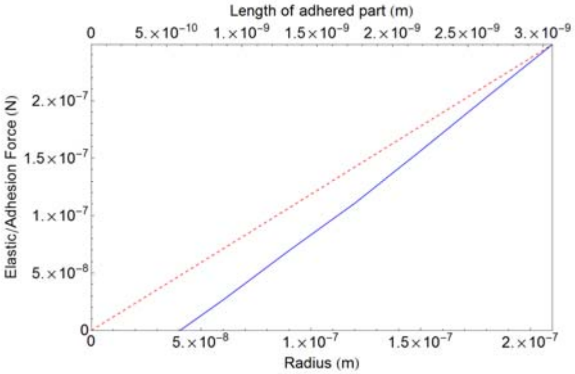

Figure S5.

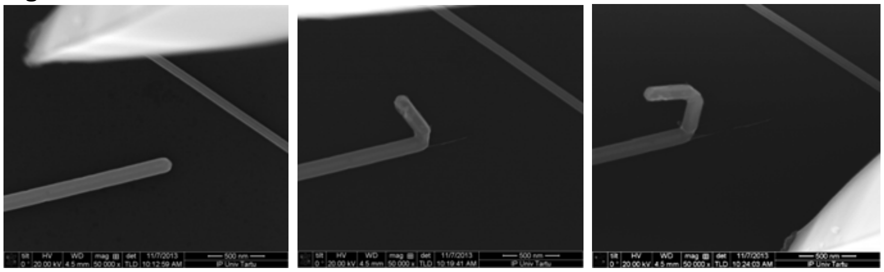

Series of SEM images demonstrating manipulation of untreated Ag NWs. It was almost impossible to displace an untreated Ag NWs on a silicon substrate without severe damage and plastic deformation of NW. After NW displacement traces are visible.

Figure S6.

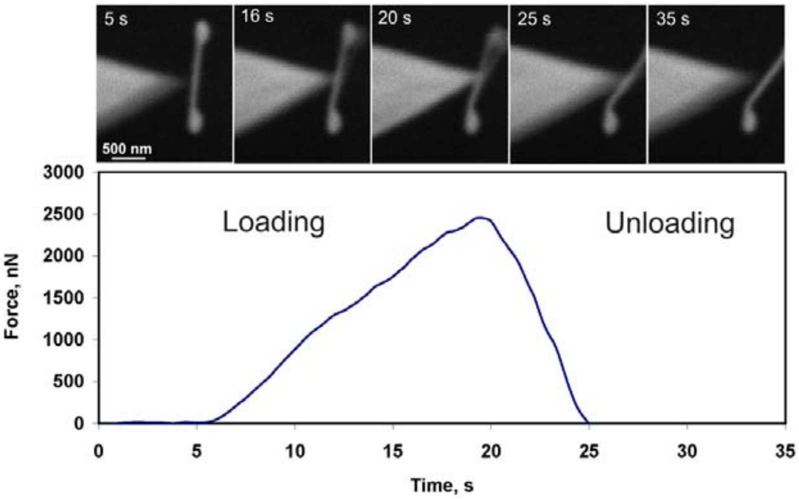

Bending of Ag ND: the series of SEM images and corresponding force curve. Young modulus determined from the force curve is 100 GPa. Details of the calculations can be found in [S. Vlassov et al. Elasticity and yield strength of pentagonal silver nanowires: in situ bending tests, *Materials Chemistry and Physics,* in press].
